# Supplementary material for: From chemoproteomic‐detected amino acids to genomic coordinates: insights into precise multi‐omic data integration
Source: Mol Syst Biol. 2021 Feb 18;17(2):e9840. doi: 10.15252/msb.20209840 (PMC7890448; doi:10.15252/msb.20209840)
Supplement: Supplementary file 1 — Appendix [file MSB-17-e9840-s001.pdf]

Appendix for:

From Chemoproteomic-Detected Amino Acids to Genomic Coordinates: Insights into Precise Multi-omic Data Integration

Maria F. Palafox[1], Heta Desai[3,5], Valerie A. Arboleda[1,2,5,7,8*], Keriann M. Backus[3,4,5,6,7,8*]

1. Department of Human Genetics, David Geffen School of Medicine, UCLA, Los Angeles, CA, 90095, USA.
2. Department of Pathology and Laboratory Medicine, David Geffen School of Medicine, UCLA, Los Angeles, CA, 90095, USA.
3. Department of Biological Chemistry, David Geffen School of Medicine, UCLA, Los Angeles, CA, 90095, USA.
4. Department of Chemistry and Biochemistry, College of Arts and Sciences, UCLA, Los Angeles, CA, 90095, USA.
5. Molecular Biology Institute, UCLA, Los Angeles, CA, 90095, USA.
6. DOE Institute for Genomics and Proteomics, UCLA, Los Angeles, CA, 90095, USA.
7. Jonsson Comprehensive Cancer Center, UCLA, Los Angeles, CA, 90095, USA.
8. Eli and Edythe Broad Center of Regenerative Medicine and Stem Cell Research, UCLA, Los Angeles, CA, 90095, USA.

*Corresponding Authors: varboleda@mednet.ucla.edu and kbackus@mednet.ucla.edu

Contents:

Appendix Table S1. Definitions of key terms

Appendix Figure S1 - Data losses that result from re-mapping chemoproteomic datasets to new releases of Ensembl and UniProtKB.

Appendix Figure S2. UniprotKB Human Proteome ID counts in cross-referenced databases.

Appendix Figure S3. Comparison of single and multi-isoform UniProtKB protein mapping to identical Ensemble protein sequences, using Ensemble xref files.

Appendix Figure S4. Comparison of single and multi-isoform UniProtKB protein mapping to identical Ensemble protein sequences, using versioned ID xref files.

Appendix Figure S5. Sequence similarity between UniProtKB protein sequences and protein sequences associated with Ensembl stable IDs across releases.

Appendix Figure S6. Comparison of GRCh37 and GrCh38 CADD models for loss of cysteine and loss of lysine.

Appendix Figure S7. Correlation of pathogenicity scores for all possible non-synonymous SNVs at codons of detected or undetected cysteine and lysine residues.

Appendix Figure S8. CADD38 PHRED scores for all possible missense variants at CpD cysteine and lysine codons, stratified by Grantham score.

Appendix Figure S9. Correlation of cysteine reactivity for residue positions found in both the 2010 CpD Cys and 2019 CpD Cys datasets

APPENDIX REFERENCES

Appendix Table S1. Definitions of Key Terms

	**Term**	**Definition**	References
**1**	Database update	Updated compilation of database resources, typically driven by gene or transcript re-annotation projects.	(Breuza *et al*, 2016; Potter *et al*, 2004)
**2**	Cross-reference	Referred to as 'xref' by UniProt and Ensembl, these files contain ID translations to equivalent sequences in other databases. These translations are what many mapping tools reference in order to translate user input.	(McGarvey *et al*, 2019; Ruffier *et al*, 2017)
**3**	Stable ID	The main citable identifier type from Ensembl and UniProtKB (primary accession). Ensembl IDs lack version number extensions (“.#”) and UniProtKB IDs lack specific isoform names (“-#”).	https://uswest.ensembl.org/info/genome/stable_ids/index.html
**4**	Canonical protein isoform ID	For UniProtKB canonical proteins, the stable ID refers to both the canonical sequence and all known protein isoforms of a given gene. Canonical protein isoform IDs display the specific isoform name of the canonical protein with a "-#" extension.	https://www.uniprot.org/help/canonical_and_isoforms
**5**	Versioned ID	Ensembl IDs with a '.#' extension, with increments to protein IDs indicating that the associated sequence has changed.	https://uswest.ensembl.org/info/genome/stable_ids/index.html
**6**	Mapping methods	1. ID mapping, translating IDs between different databases.	(Meyer *et al*, 2016; Huang *et al*, 2008)
		2. Residue-residue mapping, a one-to-one correspondence between amino acids in proteins from different databases.	(David & Yip, 2008; Martin, 2005; Dana *et al*, 2019)
		3. Residue-codon mapping, a one-to-three correspondence between an amino acid and nucleotide coordinates (codon) in a reference genome	(Li *et al*, 2016; Zhou *et al*, 2015; Stephenson *et al*, 2019)

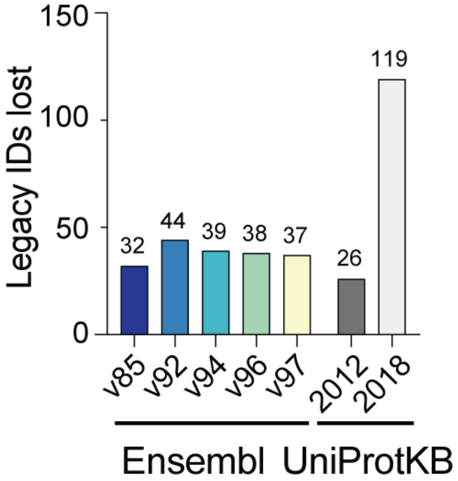

Appendix Figure S1. Data losses that result from re-mapping chemoproteomic datasets to new releases of Ensembl and UniProtKB. Shows the number of stable UniProtKB protein IDs from cysteine and lysine chemoproteomics studies in original legacy chemoproteomics dataset (4,119 Uniprot stable IDs in aggregate) (Hacker *et al*, 2017; Backus *et al*, 2016; Weerapana *et al*, 2010) that fail to map to IDs in more recent releases of Ensembl and UniProtKB. While all Ensembl datasets showed similar losses, Ensembl v85 modestly outperformed more recent versions, consistent with the v85 release date being closest in time to the UniProtKB release on which legacy data was based.

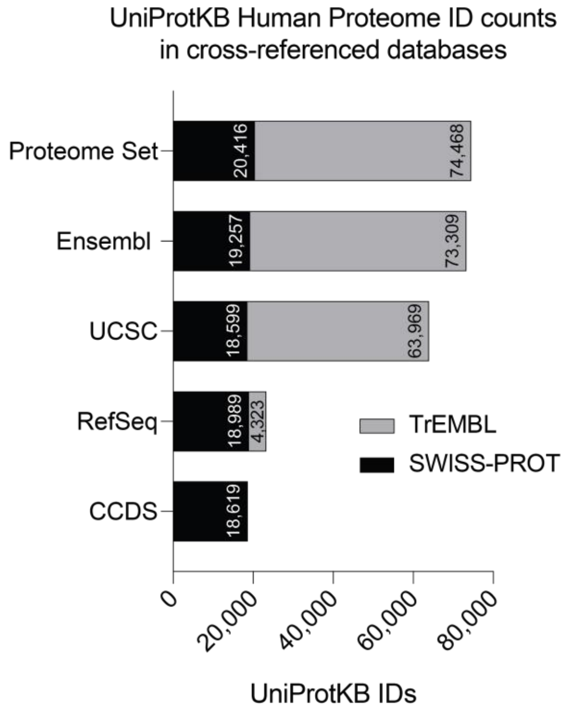

Appendix Figure S2. **UniprotKB Human Proteome ID counts in cross-referenced databases**. The UniProtKB/TrEMBL subset (automated translations of coding sequences) are shown in grey and the UniProtKB/Swiss-Prot subset (manually curated sequences) are shown in black. Ensembl, USCS, and RefSeq contain both automated (TrEMBL) and manually curated (Swiss-Prot) entries. Sequences derived from the consensus coding sequence (CCDS) project are associated with the UniProtKB/Swiss-Prot subset.

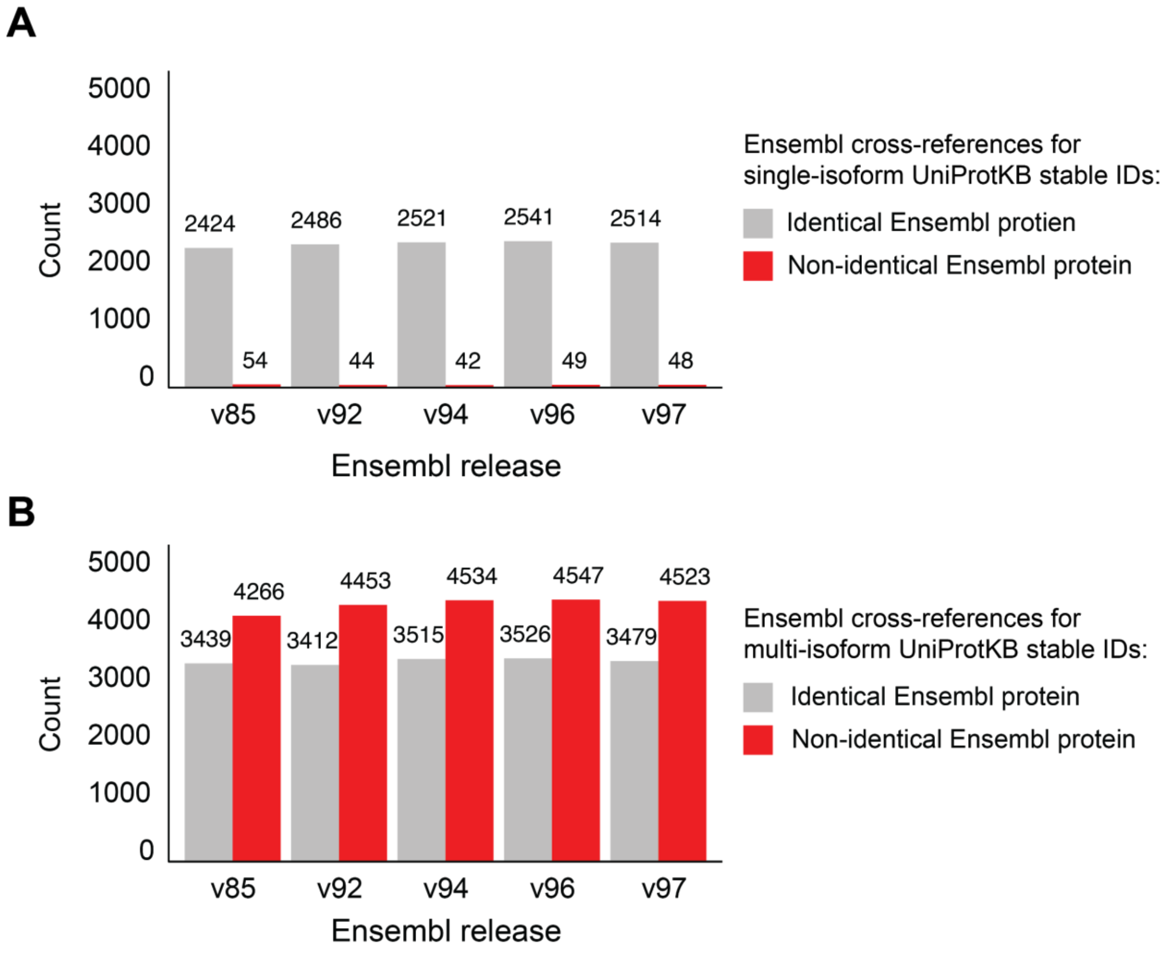

Appendix Figure S3. Comparison of single and multi-isoform UniProtKB protein cross-references to Ensembl proteins, using the Ensembl xref files. Using five Ensembl xref files (Materials and Methods, **Method A**) containing only stable ID cross-references to UniProtKB IDs, protein sequences were compared for A) 1,466 single isoform UniProKB IDs and B) 2,487 multi-isoform UniProKB IDs contained in our CpDAA-containing protein dataset.

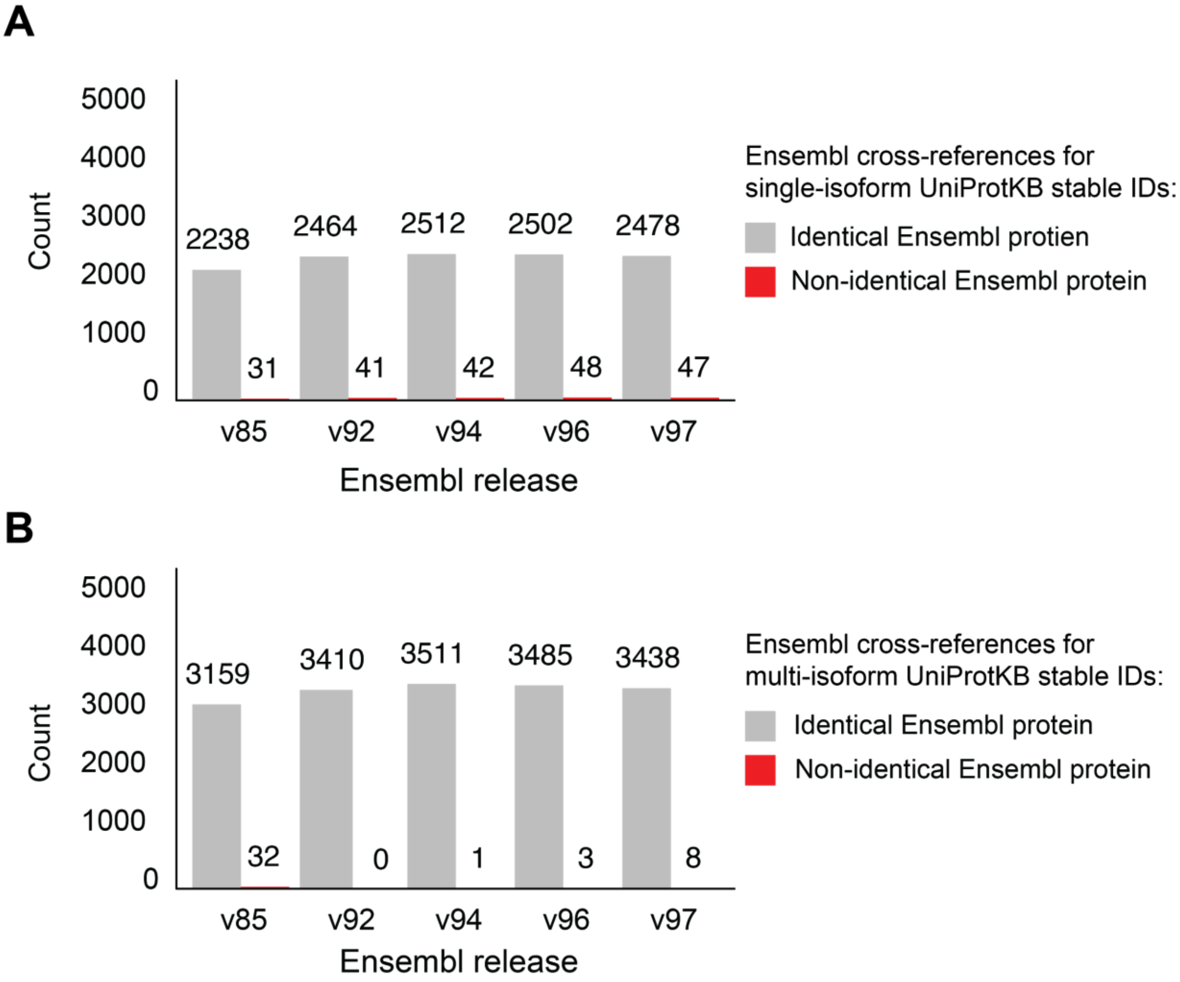

Appendix Figure S4. Comparison of single and multi-isoform UniProtKB protein cross-references to Ensembl proteins, using the UniProtKB mapping file. Using UniProtKB mapping file (Materials and Methods, **Method B**) provided canonical protein isoform ID cross-references to Ensembl stable protein IDs. Comparisons between UniProtKB canonical proteins from 2018_06 release were made to Ensembl proteins from five releases. Results of sequence identity comparison was performed for A) 1,466 single isoform UniProtKB IDs and B) 2,487 multi-isoform UniProKB IDs contained in our CpDAA-containing protein dataset.

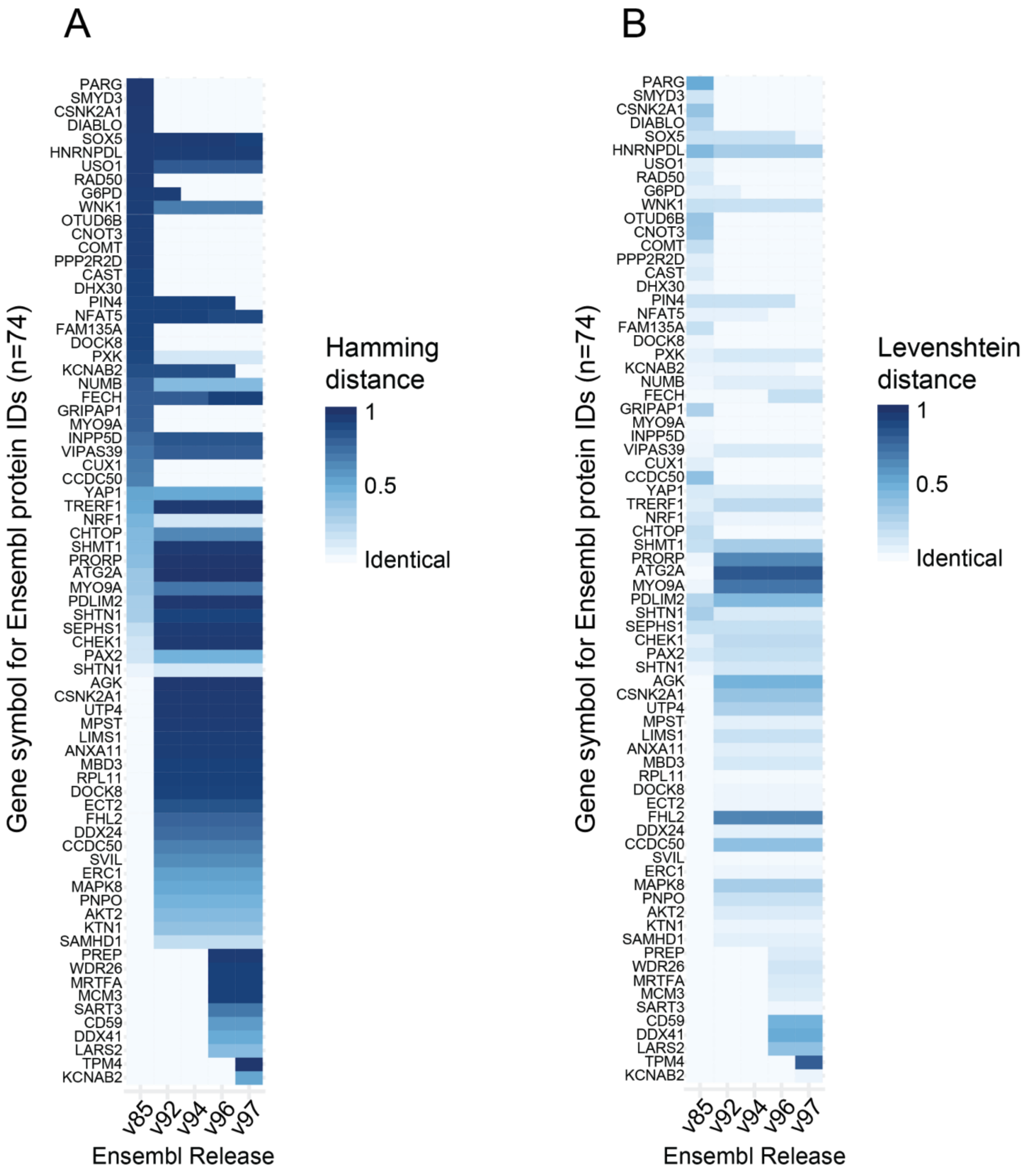

Appendix Figure S5. Sequence similarity between UniProtKB protein sequences and protein sequences associated with Ensembl stable IDs across releases. Heatmaps show A) normalized Hamming distance and B) normalized Levenshtein distance for sequence alignments of the protein sequences associated with the top 74 stable Ensembl gene, transcript, and protein IDs with an identical cross-referenced Ensembl protein sequence in one release, but non-identical sequences in additional releases. Scores range from 0 to 1, with 0 indicating identical to the

canonical sequence in the 2018 UniProtKB CCDS release. Source data is shown in **Source Data for S5 Table**, which includes the 49 UniProtKB IDs that had no canonical sequence equivalent in all five Ensembl releases analyzed and CpDAA index differences for most detected cysteine or lysine positions.

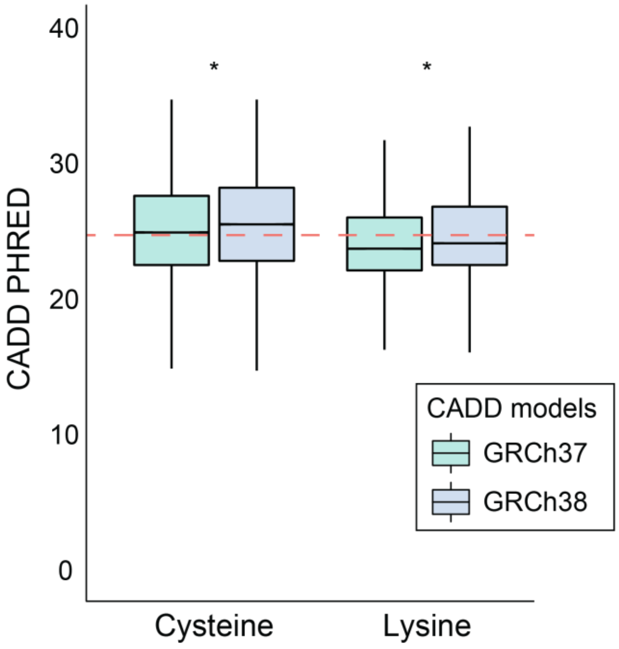

Appendix Figure S6. Comparison of GRCh37 and GrCh38 CADD models for loss of cysteine and loss of lysine. Loss of cysteine (n= 280,748) and loss of lysine (n= 1,046,638) missense overlapping coordinates of residues in 3,840 detected proteins. Deleterious missense threshold for CADD PHRED score of 25 marked by red dashed line. Cysteine missense score average is 24.34 +- 5.88 s.d. for CADD GRCh37 model (green) and 25.51 +- 5.10 s.d. for CADD GRCh38 model. Lysine missense score average is 23.55 +- 4.97 s.d. for CADD GRCh37 model and 24.70 +- 4.14 s.d. for CADD GRCh38 model. Wilcox test used for pairwise comparisons, * = p-value <2e-16 (CADD38-CADD37 PHRED score mean difference of 1.16 +- 2.56 s.d. for all cysteine and lysine residues in 3,840 detected proteins).

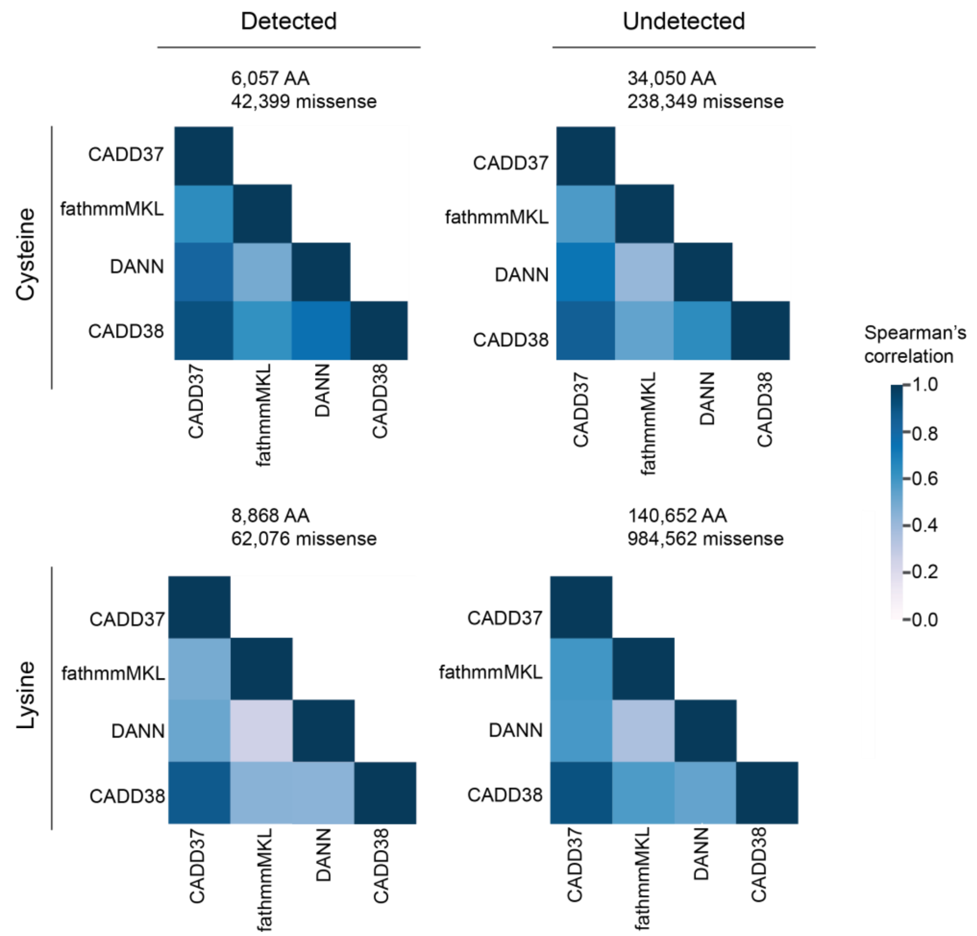

Appendix Figure S7. Correlation of pathogenicity scores for all possible non-synonymous SNVs at codons of detected or undetected cysteine and lysine residues. Heatmap represents two-tailed Spearman's rank-order correlation coefficients for all possible non-synonymous SNVs at codons of detected or undetected cysteine and lysine residues in 3,840 detected proteins. Only the subset of scores that provide pathogenicity annotations for all possible non-synonymous variants were included in this analysis.

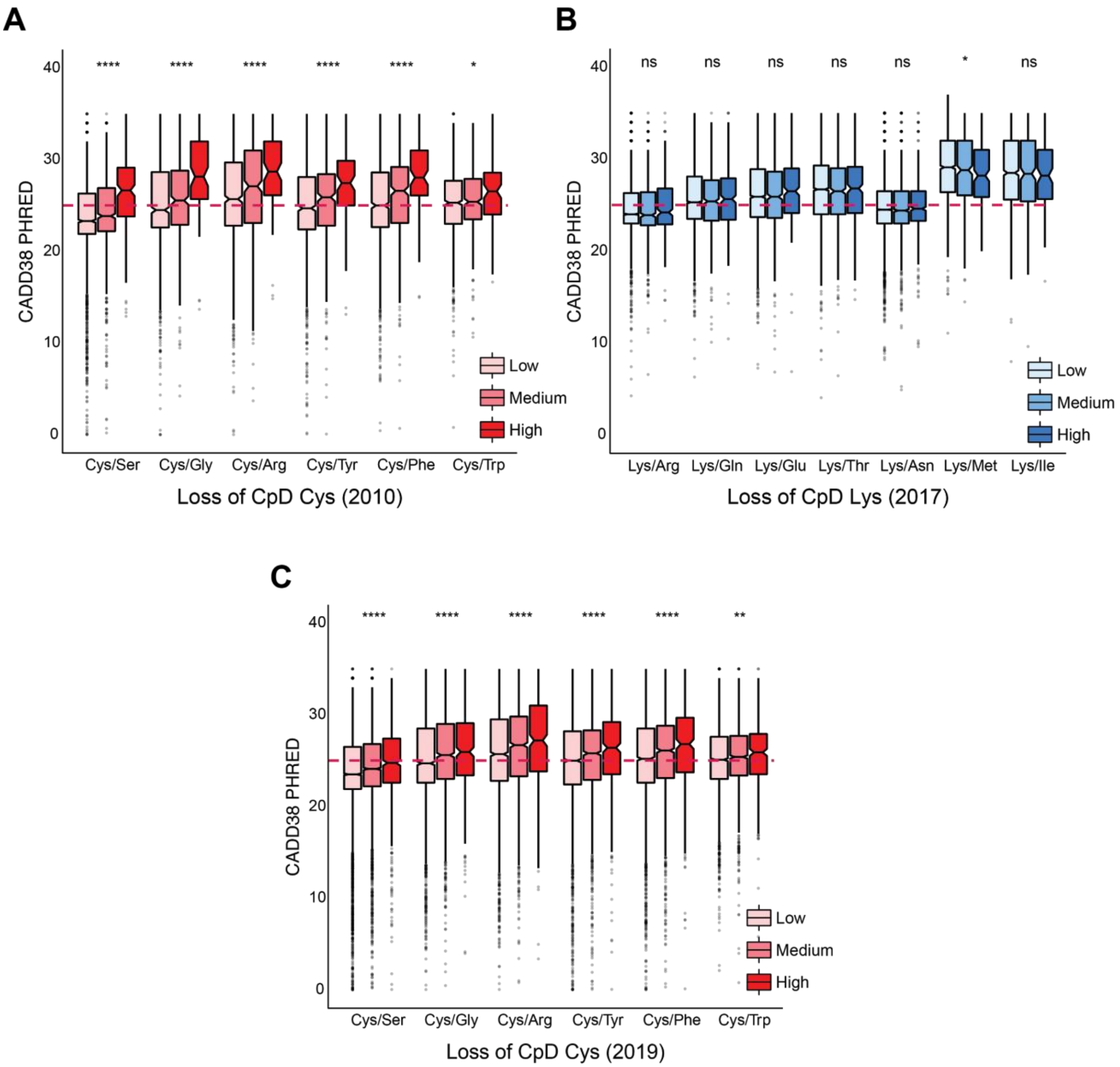

Appendix Figure S8. CADD38 PHRED scores for all possible missense variants at CpD cysteine and lysine codons, ordered by Grantham score. Distribution of CADD38 (model for GRCh38) PHRED scores for cysteine and lysine CpDAAs of Low, Medium, and High intrinsic reactivities, defined by isoTOP-ABPP ratios, Low ($R_{10:1}>5$), Medium ($2<R_{10:1}<5$), High ($R_{10:1}<2$)(Weerapana *et al*, 2010; Hacker *et al*, 2017).

A) Enrichment of predicted deleterious missense variants for highly reactive cysteine residues identified by (Weerapana *et al*, 2010). B) No enrichment of predicted deleterious missense variants for highly reactive lysine residues identified by (Hacker *et al*, 2017). C) Enrichment of predicted deleterious missense variants for highly reactive cysteine residues identified in the current study. Kruskal-Wallis nonparametric test to examine reactivity group difference, **p value* = 0.01, ***p value* = 0.003, *****p value* = <1.1e-06.

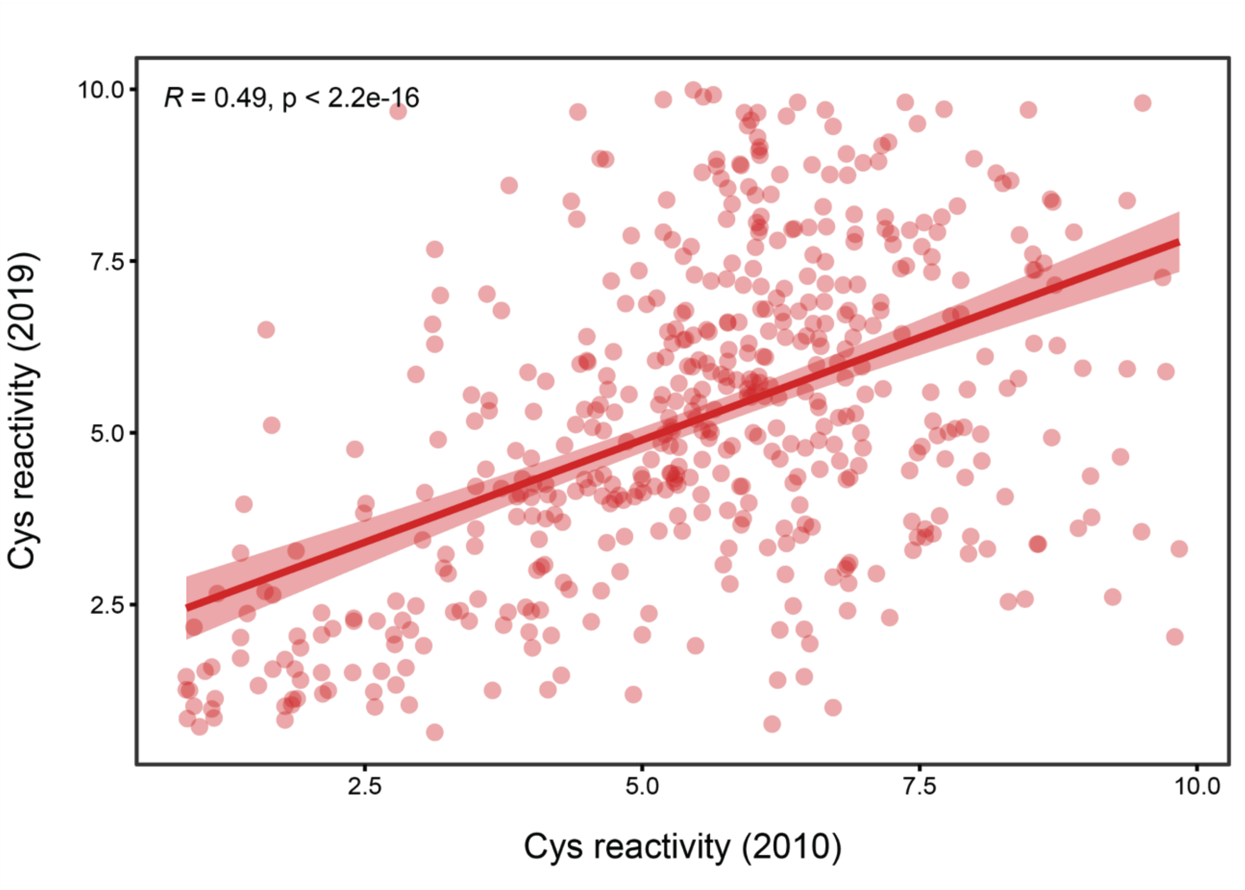

Appendix Figure S9. Correlation of cysteine reactivity between different chemoproteomic datasets. Total of 502 CpDAA are shared between the 2010 CpD Cys (Weerapana *et al*, 2010) and 2019 CpD Cys reactivity dataset (Current study; Dataset EV18). Pearson's correlation coefficient (R) = 0.49, p value < 2.2e-16, and 95% confidence interval of coefficient [0.425, 0.558].

REFERENCES

Backus KM, Correia BE, Lum KM, Forli S, Horning BD, Gonzalez-Paez GE, Chatterjee S, Lanning BR, Teijaro JR, Olson AJ, *et al* (2016) Proteome-wide covalent ligand discovery in native biological systems. *Nature* 534: 570–574

Breuza L, Poux S, Estreicher A, Famiglietti ML, Magrane M, Tognolli M, Bridge A, Baratin D, Redaschi N & UniProt Consortium (2016) The UniProtKB guide to the human proteome. *Database* 2016

Dana JM, Gutmanas A, Tyagi N, Qi G, O'Donovan C, Martin M & Velankar S (2019) SIFTS: updated Structure Integration with Function, Taxonomy and Sequences resource allows 40-fold increase in coverage of structure-based annotations for proteins. *Nucleic Acids Research* 47: D482–D489 doi:10.1093/nar/gky1114 [PREPRINT]

David FPA & Yip YL (2008) SSMap: a new UniProt-PDB mapping resource for the curation of structural-related information in the UniProt/Swiss-Prot Knowledgebase. *BMC Bioinformatics* 9: 391

Hacker SM, Backus KM, Lazear MR, Forli S, Correia BE & Cravatt BF (2017) Global profiling of lysine reactivity and ligandability in the human proteome. *Nat Chem* 9: 1181–1190

Huang DW, Sherman BT, Stephens R, Baseler MW, Lane HC & Lempicki RA (2008) DAVID gene ID conversion tool. *Bioinformation* 2: 428–430

Li Y, Wang X, Cho J-H, Shaw TI, Wu Z, Bai B, Wang H, Zhou S, Beach TG, Wu G, *et al* (2016) JUMPg: An Integrative Proteogenomics Pipeline Identifying Unannotated Proteins in Human Brain and Cancer Cells. *J Proteome Res* 15: 2309–2320

Martin ACR (2005) Mapping PDB chains to UniProtKB entries. *Bioinformatics* 21: 4297–4301

McGarvey PB, Nightingale A, Luo J, Huang H, Martin MJ, Wu C & UniProt Consortium (2019) UniProt genomic mapping for deciphering functional effects of missense variants. *Hum Mutat* 40: 694–705

Meyer MJ, Geske P & Yu H (2016) BISQUE: locus- and variant-specific conversion of genomic, transcriptomic and proteomic database identifiers. *Bioinformatics* 32: 1598–1600

Potter SC, Clarke L, Curwen V, Keenan S, Mongin E, Searle SMJ, Stabenau A, Storey R & Clamp M (2004) The Ensembl analysis pipeline. *Genome Res* 14: 934–941

Ruffier M, Kähäri A, Komorowska M, Keenan S, Laird M, Longden I, Proctor G, Searle S, Staines D, Taylor K, *et al* (2017) Ensembl core software resources: storage and programmatic access for DNA sequence and genome annotation. *Database* 2017

Stephenson JD, Laskowski RA, Nightingale A, Hurles ME & Thornton JM (2019) VarMap: a web tool for mapping genomic coordinates to protein sequence and structure and retrieving protein structural annotations. *Bioinformatics* 35: 4854–4856

Weerapana E, Wang C, Simon GM, Richter F, Khare S, Dillon MB, Bachovchin DA, Mowen K, Baker D & Cravatt BF (2010) Quantitative reactivity profiling predicts functional cysteines in proteomes. *Nature* 468: 790–795

Zhou W, Chen T, Chong Z, Rohrdanz MA, Melott JM, Wakefield C, Zeng J, Weinstein JN, Meric-Bernstam F, Mills GB, *et al* (2015) TransVar: a multilevel variant annotator for precision genomics. *Nat Methods* 12: 1002–1003
