# Supplementary material for: From chemoproteomic‐detected amino acids to genomic coordinates: insights into precise multi‐omic data integration
Source: Mol Syst Biol. 2021 Feb 18;17(2):e9840. doi: 10.15252/msb.20209840 (PMC7890448; doi:10.15252/msb.20209840)
Supplement: Supplementary file 2 — Expanded View Figures PDF [file MSB-17-e9840-s002.pdf]

Expanded View Figures

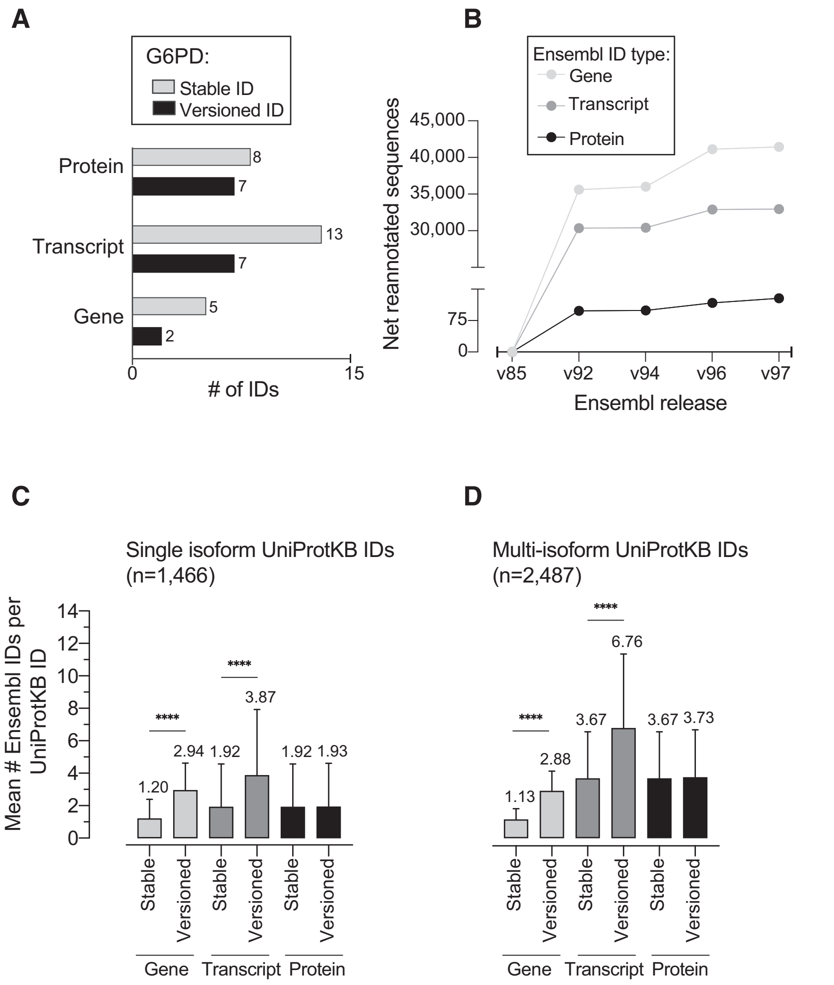

Figure EV1. Mapping of Ensembl IDs to UniprotKB shows heterogeneity at gene, transcript, and protein levels.

A Number of stable and versioned Ensembl gene, transcript, and protein IDs for G6PD across all five Ensembl releases.

B Cumulative sequence re-annotations for Ensembl gene, transcript, and protein IDs since the v85 release.

C, D Average number of Ensembl gene, transcript, and protein IDs for (C) single isoform (n = 1,466) and (D) multi-isoform (n = 2,487) CpDAA UniProt entries. Bar plots represent mean values ± SD for the number of Ensembl IDs per stable UniProtKB ID. Statistical significance was calculated using an unpaired Student's t-test, ****P-value < 0.0001.

 © 2021 The Authors

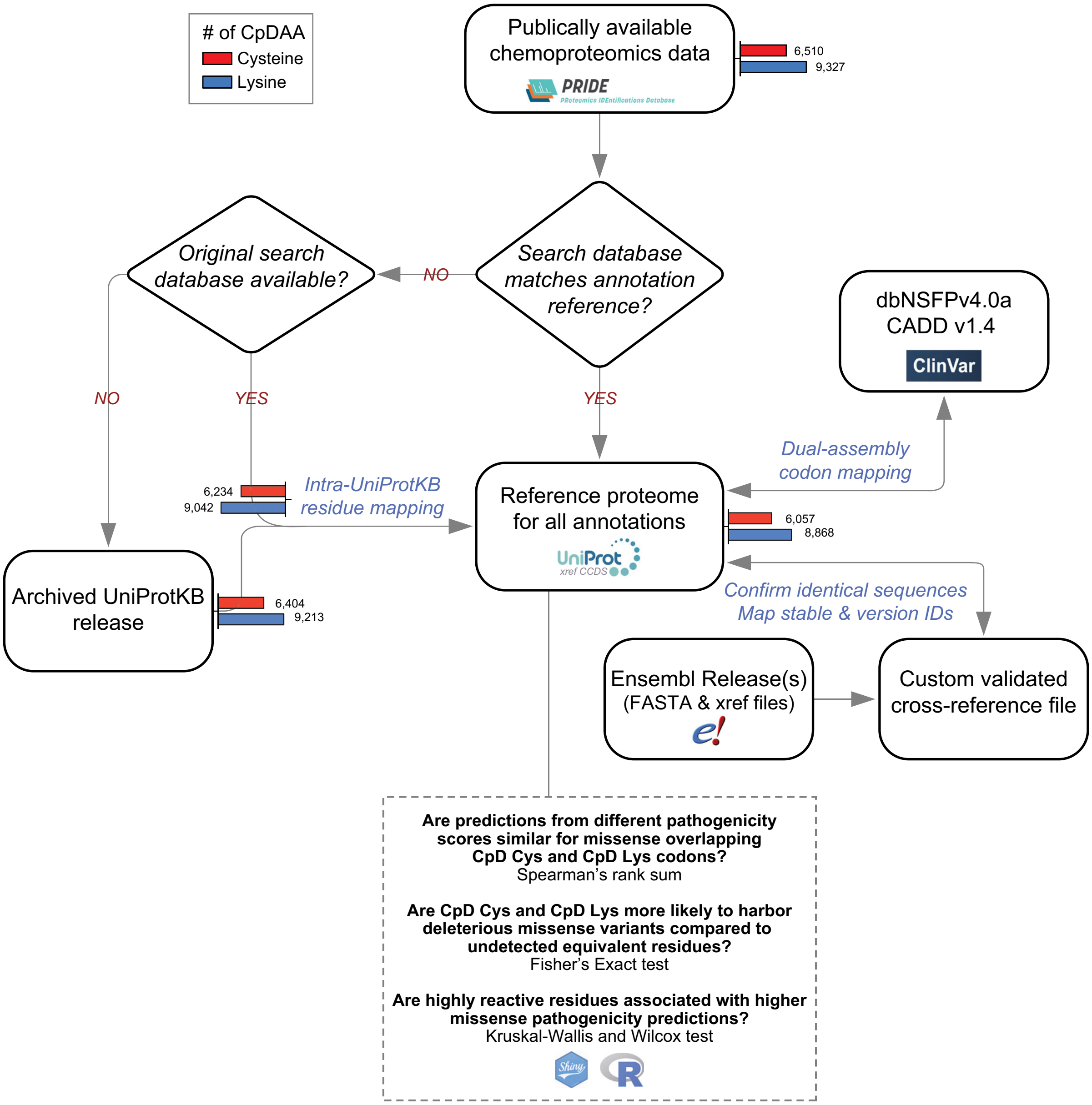

Figure EV2. Flowchart of the mapping strategy and data analysis.

CpD cysteines and lysines from three publicly available datasets were processed and filtered according to our optimized mapping pipeline. Number of CpD cysteines (red) and CpD lysines (blue) retained following each step shown as bar plots.

© 2021 The Authors

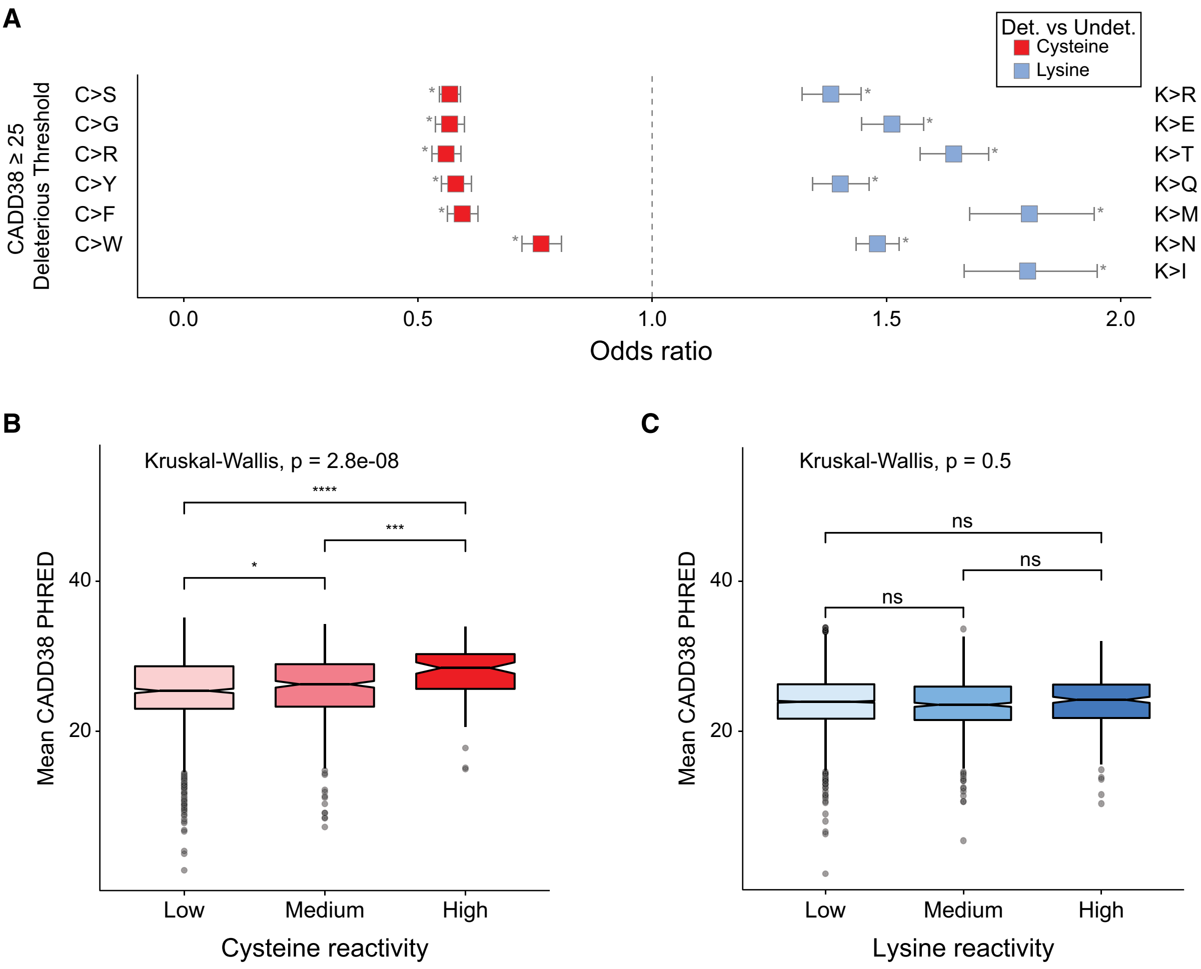

Figure EV3. Assessment of missense pathogenicity between detected–undetected and reactivity groups for CPD cysteine and lysine residues.

A Enrichment of predicted deleterious missense variants for detected vs undetected cysteine (red) and lysine (blue) missense variants in 3,840 proteins. Missense types in order of increasing Grantham score. Odds ratio (OR) for all possible nonsynonymous SNVs at cysteine codons between 0.56 and 0.76, at lysine codons fall between 1.38 and 1.80. 95% confidence intervals (line segments) and odds ratios (squares), two-tailed Fisher's exact test, *P < p cut-off, and 0.0019 Bonferroni-corrected (0.05/26).

B, C Distribution of mean CADD38 (model for GRCh38) PHRED scores for (B) cysteine (n = 1,401) and (C) lysine (n = 4,363) CpDAAs of low, medium, and high intrinsic reactivities, defined by isoTOP-ABPP ratios, low ($R_{10:1} > 5$), medium ($2 < R_{10:1} < 5$), high ($R_{10:1} < 2$) (Weerapana *et al*, 2010; Hacker *et al*, 2017). Kruskal–Wallis nonparametric test to examine reactivity group difference, Wilcox test used for pairwise comparisons (BH-adjusted P-values, *P. adj = 0.013, ***P. adj = 2.80e-05, and ****P. adj = 5.30e-08). The boxplot boxes represent the lower and upper quartiles, with the central band as median, notches show the confidence interval based on median ± 1.58*IQR/sqrt(n), and whiskers mark observations that satisfy quartiles ± 1.5*IQR.

 © 2021 The Authors

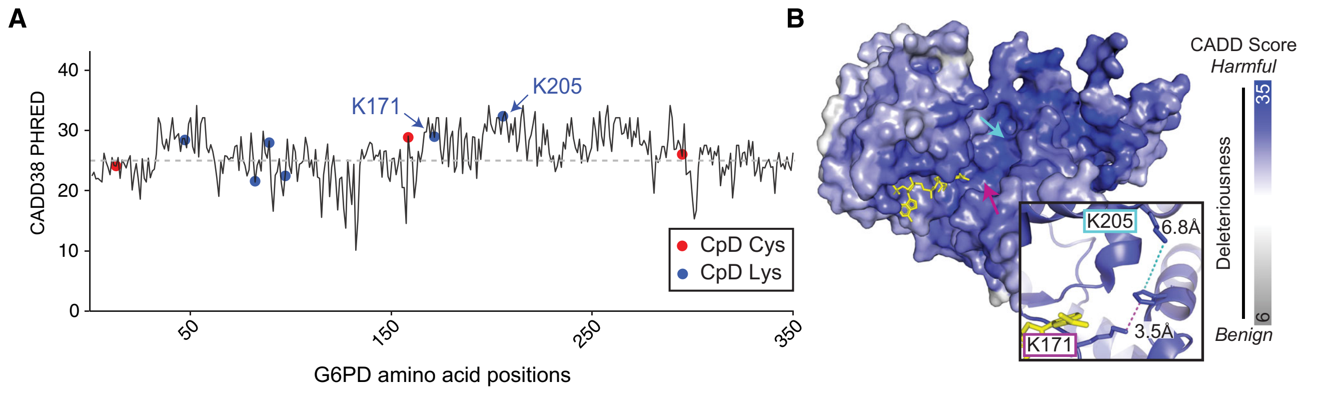

Figure EV4. Functional validation of reactive lysine in G6PD.

A Shows CADD38 max codon missense scores for residues 1–350 of G6PD (UniProt ID P11413). CpD K205 has the highest score out of all positions in protein. CpDAA positions above CADD38 deleterious threshold (gray dash line) include K47, K89, C158, K171, K205, and C294.

B Crystal structure of G6PD (PDB ID: 2BH9) shows K205 and K171 located within the enzyme active site. NADP^{+} cofactor shown in yellow. Surface colored by CADD38 max codon missense scores. Image generated in PyMOL (Smith *et al*, 2019).

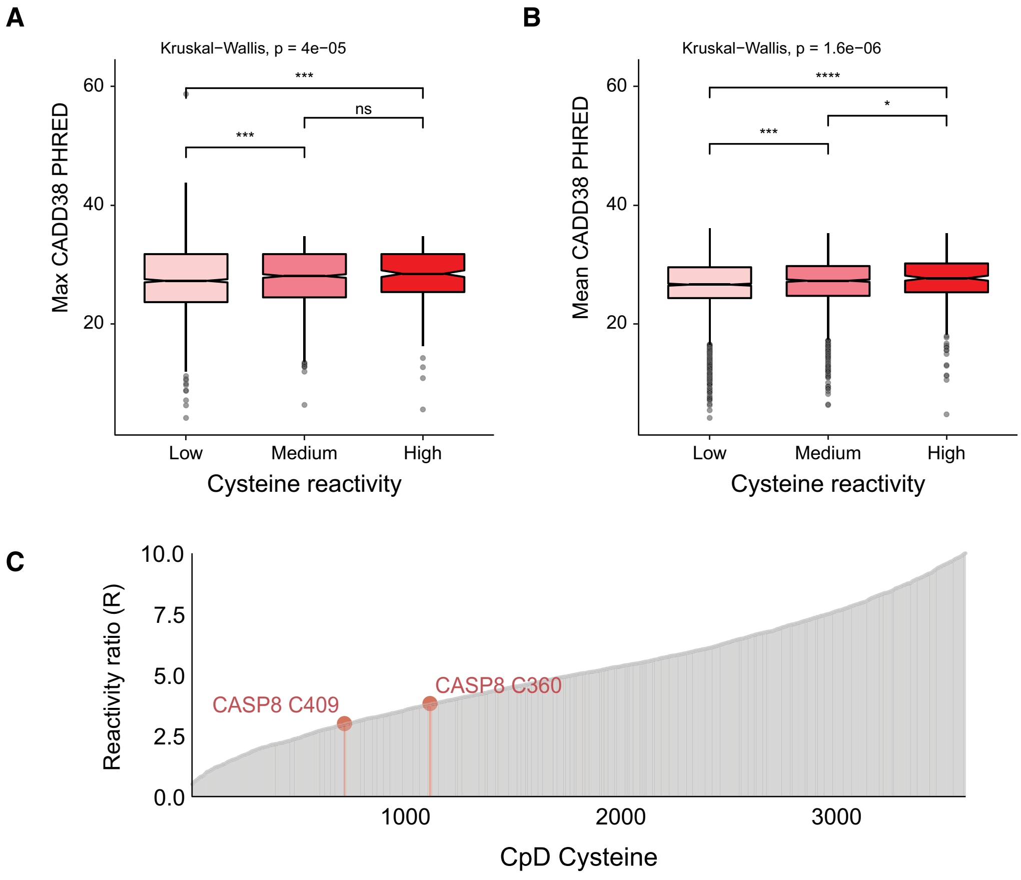

Figure EV5.

Figure EV5. 2019 Cysteine chemoproteomics data support residue reactivity and deleteriousness score trend.

A, B Association between cysteine reactivity labels and CADD38 (model for GRCh38) PHRED scores for cysteines of low (n = 2,247), medium (n = 1,448), and high (n = 322) intrinsic reactivities, defined by isoTOP-ABPP ratios, low ($R_{10:1} > 5$), medium ($2 < R_{10:1} < 5$), high ($R_{10:1} < 2$) (Weerapana *et al*, 2010; Hacker *et al*, 2017). Either the max CADD score for a missense change was assigned to the codon (BH-adjusted *P*-values, low vs med ***P. adj = 0.00099, low vs high ***P. adj = 0.00086) (A) or the average of all missense scores at that codon (BH-adjusted *P*-values, low vs med ***P. adj = 4.0e-04, med vs high *P. adj = 0.023, low vs high ****P. adj = 3.90e-05) (B). Reactivity group differences assessed by Kruskal–Wallis nonparametric test and Wilcox test used for pairwise comparisons.

C Plot of cysteine reactivity ratios for 3,590 out of 4,017 total profiled residues in 2019 isoTOP-ABPP study. Represented are 322 high, 1,448 medium, and 1,820 low threshold cysteines.

© 2021 The Authors
